# Flammability of Two Mediterranean Mixed Forests: Study of the Non-additive Effect of Fuel Mixtures in Laboratory

**DOI:** 10.3389/fpls.2018.00825

**Published:** 2018-06-25

**Authors:** Gianni Della Rocca, Roberto Danti, Carmen Hernando, Mercedes Guijarro, Javier Madrigal

**Affiliations:** ^1^Institute for Sustainable Plant Protection-National Research Council of Italy, Sesto Fiorentino, Italy; ^2^Department of Silviculture and Forest Management, INIA–CIFOR, Madrid, Spain; ^3^Instituto Universitario de Investigación en Gestión Forestal Sostenible, UVa-INIA, Madrid, Spain

**Keywords:** live foliage, litter, bark, oaks, Cupressaceae, fire resilience, ignition, mixed forests

## Abstract

In the Mediterranean region, wildfires are a major disturbance, determined by ecosystem and forest species characteristics. Both the flammability and resistance to fire of a mixed forest may vary from those of the individual species. Two mixed Mediterranean woodlands, a *Cupressus sempervirens* and *Quercus ilex* stand in Italy; and a *Juniperus thurifera* and *Quercus faginea* stand in Spain were investigated. Laboratory flammability tests were conducted on live foliage, litter samples and on litter beds from individual and mixed species to evaluate: (i) the flammability traits of the mixtures of live foliage and litter samples; (ii) whether the flammability of the two-species mixtures are non-additive, i.e., differ from expected flammability based on arithmetic sum of the single effects of each components species in monospecific fuel; (iii) the ignition success and initial fire propagation in litter beds. Flammability tests were also conducted on bark samples to estimate the resistance of the tree species to fire. The ignitibility of live foliage was lower and the combustibility was higher in *Cupressaceae* than in *Quercus*. Non-additive effects were observed in some flammability components of live foliage and litter, especially in the mixtures of *C. sempervirens* and *Q. ilex*. Ignitability and combustibility were higher and lower than expected, respectively, and tended to be driven by *Quercus*), while the consumability was lowered more than expected by both *Cupressaceae*. The ignition success in the litter beds was low, especially for the presence of *Cupressaceae* that increase the bulk density of the mixtures. *Cupressaceae*, which have a thinner bark, suffered more damage to the cambium after shorter exposure to the heat source than *Quercus* species. In all the species studied, time to reach lethal temperatures in the cambium was dependent on thickness rather than on flammability of the bark. The study findings revealed that tree species may influence flammability of mixed fuels disproportionately to their load. The studied species showed to exert a contrasted effect on flammability of the mixtures, increasing ignitability and decreasing combustibility and consumability well out of their proportion in the mixture. This may potentially influence fire dynamics in mixed forests.

## Introduction

Mixed forests may be more resistant and resilient than monospecific forests to anthropic and environmental disturbance, with a more intense and quicker response (Loreau et al., 2001; Jactel et al., 2009). Moreover they generally have higher biodiversity levels and higher carbon storage capacity. This is reflected in better adaptation to global change, greater potential for mitigation strategies (Wagner et al., 2014), higher water and nutrient regulation and higher productivity and support for ecosystem services than in monospecific woodlands (Knoke et al., 2008).

Recurrent wildfires are one of the main causes of forest degradation, especially in the Mediterranean area (Fares et al., 2017). Moreover, fire is a powerful ecological factor shaping vegetation distribution and structure across many biomes (de Magalhães and Schwilk, 2012). Information about the effects of wildfires on mixed and monospecific forests, i.e., different degree of damages caused, different mortality of species, rate of fire spread, duration and intensity that are affected by the stand composition could provide a better knowledge of forest management strategies aimed at fire control and prevention.

The relationship between the incidence of forest fires and type of woodland is not clear (Vázquez de la Cueva et al., 2012, 2015; Pereira et al., 2014). Fire preference in relation to land cover depends on several potential drivers such as land cover distribution and fuel availability, climatic and meteorological conditions, fire regime, and topographic features (Barros and Pereira, 2014; Oliveira et al., 2014). Some studies conducted in central and southern Europe have shown that stand composition was the most important variable explaining fire probability. [Pezzatti et al., 2009; Bajocco et al., 2011; In Southern Europe (Portugal), the effect of fire size on selectivity is clear for most forest types (conifer forests, plantations and shrubland), but less clear for annual crops and evergreen open oak woodlands Moreira et al., 2009; Silva et al., 2009; Barros and Pereira, 2014].

Mixed forests are characterized as having the lowest proportion of burnt area of the forestland in Southern Europe, but as being particularly prone to fire in relation to the total covered area (Pereira et al., 2014). Special attention should therefore be given to forest management and fire prevention in this type of forest (Pereira et al., 2014). The definition of fire risk in mixed forest and assessment of the different components driving the risk could lead to the development of silvicultural recommendations and prescriptions.

Characterization of forest fuel flammability, interactively driven by many dynamic factors, is a basic step in fire hazard assessment. Indeed, ignition, fireline intensity, rate of spread, and fuel consumption result from the interactions between fuel characteristics such as species composition, canopy architecture, moisture content, and wind speed (Fernandes and Cruz, 2012; Varner et al., 2015).

The variation in plant flammability evaluated at fine fuel level (leaves, distal twigs, and litter) in relation to leaf morphology or chemical composition (i.e., lignin, terpenoids, and mineral plant contents) has been investigated for various species (e.g., Alessio et al., 2008a,b; De Lillis et al., 2009; Courty et al., 2012; Ganteaume et al., 2013; Cornwell et al., 2015; Pausas et al., 2016; Dehane et al., 2017). However, in natural systems, fuels often consist of a mixture of materials from various species. Furthermore, types of vegetation differ in their contribution to fuel continuity and load, and this diversity in vegetation composition and structure greatly affects fire regimes (Brooks et al., 2004). The effect of species mixture on forest fuel flammability has received scant attention and has particularly focused on litter (Ormeño et al., 2009; de Magalhães and Schwilk, 2012; van Altena et al., 2012) and ground fuels (van Altena et al., 2012; Blauw et al., 2015). Some authors have argued that the flammability of plant tissues is increased by the volatile organic compounds (VOCs) they contain (Ormeño et al., 2009; Pausas et al., 2016), possibly explaining the relatively lower flammability of species such as *Quercus* spp., which do not store isoprenoids, compared to isoprenoids-storing species (Della Rocca et al., 2017). Nevertheless, other studies have shown that the flammability of *Quercus* and conifers were quite similar (Madrigal et al., 2011; Molina et al., 2017).

Litter of *Pinus* species are frequently characterized by lower bulk density than that of *Quercus* species, which increases its flammability (Guijarro et al., 2002). By contrast, other conifers such as *Cupressus* spp. produce litter with a relatively high bulk density that is more combustible but less ignitable than Mediterranean pines (Della Rocca et al., 2015). Similar physical properties have been observed in other species of *Cupressaceae* such as *Chamaecyparis* sp. (Pinto and Fernandes, 2014), *Thuja* sp. (Molina et al., 2017), *Juniperus phoenicea* L. and *J. oxycedrus* L. The latter two species have also been found to have non-adapted fire traits (Pausas and Verdú, 2005). On the other hand, although *Quercus* species are characterized by good resprouting after fire (Pausas and Verdú, 2005), they do not have other fire-adapted traits (e.g., thick bark), except for *Q. suber* L., that would help them to resist intermediate or low intensity fires (Catry et al., 2012).

Natural mixed woodlands of *Cupressus sempervirens* L. var. *horizontalis* (Mill.) Aiton-*Quercus ilex* L. subsp. *ilex* in central Italy (43°50,1 N, 11°14,7 E) and of *Juniperus thurifera* L.-*Quercus faginea* Lam. in central Spain (40°56,1 N, 2°07,3 W) are Mediterranean types of forest characterized by combinations of conifer and broadleaved species. These woodlands are not very widespread and therefore deserve special attention in terms of conservation. Another interesting trait of these forests is the contrasting flammability of the species involved. *Cupressaceae* are reported (in laboratory studies) as being slow to ignite but develop high energy (Della Rocca et al., 2015), whereas several Mediterranean oak species, belonging to the ‘fast flammable’ group as defined by Pausas et al. (2017), generally burn more rapidly but release less heat. It would therefore be interesting to evaluate the occurrence of non-additive effects of the fuel mixtures on the flammability components, as has been done for litter (van Altena et al., 2012). An effect is non-additive when it is different from that expected based on arithmetic sum of the single effects of each components; which means that one species prevails over the other with respect to flammability (enhanced dominance) (van Altena et al., 2012). Ultimately, flammability of a mixture of species may not be predicted as the mean flammability of the single species of the mixtures (de Magalhães and Schwilk, 2012).

During a wildfire, flaming or glowing particles of fuel dispersed by wind or air turbulence can cause random spot fires outside the perimeter of the primary wildfire. This phenomenon is related to the capacity of the litter to ignite when embers land on it and mainly depends on the species generating the litter bed and consequently on the litter traits (Ganteaume et al., 2009). Spot fires are closely related to extreme fire behavior and have important consequences on the forest fire-fighting strategies (Potter, 2011). The effects of litter bulk density, moisture and species from which the litter is derived, as well as the effects of ember traits were investigated by Ganteaume et al. (2009). However, the ability of the litter derived from different species to ignite and propagate a fire relative to that of litter derived from a mixture of species (as in mixed forest) is poorly studied (van Altena et al., 2012).

The survival of trees of different species after the passage of a fire also influences the maintenance of the composition of mixed forests. The resistance of tree trunks to wildfire depends on several factors and is influenced by both fire type and species-dependent trunk characteristics, which interact and determine the fire severity (Pausas, 2015, 2017). Living tissues in the inner bark, including the cambium, are the most sensitive to fire (Frejaville et al., 2013), and the sensitivity of the trunk to fire therefore depends on the flammability and thermal conductivity of the bark (Dehane et al., 2015). Bark thickness is a key adaptive factor affecting the resistance of trees to fire (e.g., Catry et al., 2012; Pausas, 2015, 2017). At the individual tree level, the ability to survive fire depends on the degree of protection of the living tissues underlying the outer bark.

Many studies have focused on characterizing the flammability of live foliage and litter of single species by comparing or classifying them (e.g., Dibble et al., 2007; Alessio et al., 2008a; Hachmi et al., 2011; Madrigal et al., 2011; Ganteaume et al., 2013; Possell and Bell, 2013; Santana and Marrs, 2014; Grootemaat et al., 2015; Molina et al., 2017; Dehane et al., 2017). Fewer studies have aimed to measure, in laboratory studies, the non-additive effects on the flammability of litter mixtures (Ormeño et al., 2009; Curt et al., 2011; de Magalhães and Schwilk, 2012; van Altena et al., 2012). Moreover, none of these studies took into account the heat released by the burning litter in order to characterize flammability (Schwilk, 2015), and very few studies tested the effect of species on flammability of mixtures of live foliage (van Altena et al., 2012; Blauw et al., 2015).

In addition, information about the relation between flammability and thermal conductivity of bark is scarce (Dehane et al., 2015), although important for characterizing the vulnerability of trunk living tissues during wildfires (Frejaville et al., 2013).

As flammability and resistance to fire of a mixed forest may vary from those of the individual species, our work was directed to characterize the flammability of two Mediterranean mixed forest fuels, by assaying live foliage, litter beds and bark samples, and evaluating the occurrence of non-additive effects. Mixtures of the considered species have never been assessed in flammability studies so far. In particular, our study conducted in laboratory on fine fuels investigated: (i) the flammability of different mixtures of live foliage and litter samples from two mixed Mediterranean woodlands (Cupressus sempervirens and Quercus ilex subsp. Ilex; Juniperus thurifera and Quercus faginea), simulating crown and surface fire conditions; (ii) the occurrence of if non-additive effects (van Altena et al., 2012; de Magalhães and Schwilk, 2012) on the flammability of the mixtures. (iii) the success of ignition and initial fire propagation on both pure and mixed litter beds in spotting ignition tests; and (iv) tree fire resistance measured by simulating heat transfer during fire in flammability tests on the bark.

## Materials and Methods

### Study Areas and Sampling

The forest fuel samples were collected in September 2014 (live foliage and litter) and September 2015 (trunks) in an uneven aged mixed forest of *Cupressus sempervirens* var. *horizontalis*, (*Cs*) and *Quercus ilex* subsp. *ilex* (*Qi*) in the Monte Morello area, Province of Florence (Italy) and in an open mixed forest of *Juniperus thurifera* L. (*Jt*) and *Quercus faginea* Lam. (*Qf*) in the province of Guadalajara (Spain) (**Supplementary Table A1**). To our knowledge, neither of these areas has been affected by wildfires for at least 50 years. The structures of the stands were very different in the two types of woodland (**Supplementary Figure A1**), although the shape of the foliage of the representative species was similar (i.e., *Cs* similar to *Jt* and *Qi* similar to *Qf*) (**Supplementary Figure A2**).

About 2 kg of live foliage (twigs both green and woody Ø < 0.6 cm with attached leaves) of each species was separately sampled at different crown heights from 20 trees, randomly chosen in both types of mixed forest. The samples were transported to the INIA-CIFOR laboratory in hermetically sealed plastics bags, on dry ice, within 24 h of collection. The samples of each species were stirred in the laboratory to obtain representative, homogenous stocks of fuel material. The fuel moisture content (FMC) of the live foliage was promptly determined, as difference between fresh and dry weight, on an oven-drying basis at 100 ± 2°C for 24 h on four-30 g subsamples of each species. The remaining samples of live foliage were stored in a refrigerated chamber (at 4°C) and processed within 5 days.

In each mixed woodland, 20 samples (total weight of about 2 kg) of monospecific litter (leaves and pieces of both fruits and woody branches, size < 6 mm) were collected from the ground below 20 groups of trees of a same species. The litter samples were collected with the aid of iron sampling grids (40 cm × 40 cm), and each sample was placed in a plastic bag and transported to the laboratory. The litter load (kg/m^2^) and the bulk density (kg/m^3^) were determined. The latter was obtained combining the thickness of the litter layer measured in the field (in 3 points within the 40 × 40 cm grid) with litter mass. The litter samples of each species were mixed to obtain representative, homogenous fuel stocks. Parts of these stocks were stored in a refrigerated chamber (at 4°C) and used in flammability tests, within 5 days. For each species, the FMC of the litter was measured before the flammability tests were carried out, as described for live foliage.

The remaining litter samples were then stored under controlled conditions (air-conditioned chamber at 20°C and 50% relative humidity) until the FMC stabilized at around 10–12%, as determined on oven-dry basis at 100 ± 2°C for 24 h and used for point-ignition and linear fire propagation tests (see below).

Stems (diameter approximately 40 cm) of 4 mature trees of each species, cut for silvicultural activities in the 2–3 preceding weeks, were obtained in both types of mixed forest. From these samples, 10 cm thick disks were cut at 1.30 m above ground (diameter at breast height) with bark intact. The disks were used to produce from 5 to 10 portions (sapwood + bark) (10 cm × 10 cm × 10 cm) of the dimensions required for the flammability tests. The samples were then stored for 4 weeks under controlled conditions as described above (Dehane et al., 2015), until being processed. The characteristics of the stem sample are summarized in **Table 1**.

**Table 1:** Characteristics of the sapwood (+ bark) samples (10 × 10 × 10 cm) of *C. sempervirens, Q. ilex, J. thurifera and Q. faginea* tested in this study.

	FMC of wood % (dry-weight)	FMC of bark % (dry-weight)	Sample thickness (wood + bark) cm	Bark thickness mm
*Cupressus sempervirens*	10.1 ± 0.3	11.4 ± 0.2	6.12 ± 0.2	4.0 ± 0.3
*Quercus ilex*	10.2 ± 0.1	11.1 ± 0.2	5.4 ± 0.3	16.4 ± 0.8
*Juniperus thurifera*	10.0 ± 0.1	11.2 ± 0.2	5.73 ± 0.1	5.8 ± 0.1
*Quercus faginea*	10.0 ± 0.2	9.5 ± 0.1	4.56 ± 0.3	9.0 ± 0.1

*Four trees per species and from 5 to 10 sapwood sample were studied.*

### Flammability Experiments

**(a) Mass Loss Calorimeter Flammability Tests on Live foliage and Litter**

An adapted Mass Loss Calorimeter (MLC) device was used, as reported in previous studies on the flammability of forest fuels (Madrigal et al., 2009, 2011, 2013; Della Rocca et al., 2015; Dehane et al., 2017). Tests were performed using the MLC arranged in the standard horizontal configuration, according to criteria established by White and Zipperer (2010) to determine the main flammability descriptors (ignitability, sustainability, combustibility, and consumability). A porous holder (10 × 10 × 5 cm) was used to allow natural diffusion of air through the samples during tests at MLC. The MLC tests were conducted at 50 kW/m^2^, simulating severe fire conditions (Cruz et al., 2011). The dry mass of the samples was fixed at 10 g (determined with a Computrac MAX^®^ 2000XL moisture analyzer, Arizona Instrument LLC) in order to reduce the variability in results due to differences in water content (Madrigal et al., 2013). At the end of each test, the residual mass fraction (RMF) was determined with a precision balance (Mettler AB104-S).

**FIGURE 1:**
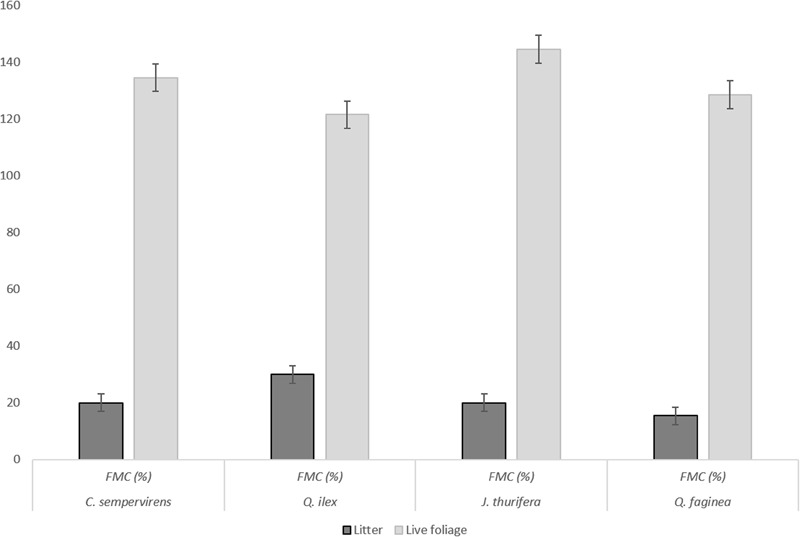
FMC (%) of the samples used in the MLC-based flammability tests, for live foliage and litter samples.

For both live foliage and litter, the MLC flammability tests were first performed on pure samples of each species and then on mixtures of *C. sempervirens–Q. ilex* and *J. thurifera-Q. faginea* (for both live foliage and litter) prepared with different proportions of the two species: 50–50%; 25–75%, and 75–25% (g/g of fresh weight). Samples were placed inside the holder so as to maintain as much as possible the natural arrangement of twigs and leaves. FMC values of live foliage and litter samples, corresponding to field moisture conditions, are shown in **Figure 1**.

The measured or calculated parameters for the different flammability phases are listed in **Table 2**. Between three and seven tests were performed for each species and mixtures to yield three replicates that complied with repeatability criteria (Madrigal et al., 2013).

**Table 2:** List of flammability components, their definitions (as in Anderson, 1970 and Martin et al., 1994) and the flammability tests performed in this study.

Experiment/Method	Component/Parameter	Variable	Units
(a) MLC: Live foliage and Litter	Ignitability	Time-to-Ignition (TTI)	s
	Combustibility	Peak heat release rate (PHRR)	kW/m^2^
	Sustainability	Total heat release (THR)	MJ/m^2^
		Average effective heat of combustion (AEHC)	MJ/kg
	Consumability	Mass loss rate (MLR)	g/s
		Residual mass fraction (RMF)	%
(b) Fire bench: Litter	(b1) Ignitability	Ignition success (IPe and IFL)	%
	(b2) Sustainability	Fire propagation (PFL)	%
	(b1) y (b2) Consumability Sustainability	Mass consumption	%
(c) MLC: Bark	Ignitability	Time-to-Ignition (TTI)	s
	Sustainability	Flame duration (FD)	s
	Combustibility	Peak heat release rate (PHRR)	kW/m^2^
	Fire resistance	Time to reach 60° C in the inner bark (TT60)	s

**(b) Fire Bench Flammability Tests on Litter beds**

The litter bed tests were conducted on a “fire bench,” on which the fuel beds were laid, forming square fuel layers of 40 cm × 40 cm (**Supplementary Figure A3**). The fire bench was placed on a scale (sensitivity 1 g) connected to a computer, enabling continuous recording of the weight loss during combustion of the fuel bed.

Two types of tests were carried out on litter samples with this device: Point-ignition source (b1) and Linear fire propagation (b2).

**(b1)** Point-ignition source tests were performed as in Ganteaume et al. (2009). Single species (pure) litter beds were artificially reconstituted (on the basis of the fuel load and bulk density of the litter measured on the field) on the fire bench. Similarly, mixed (50–50%: g/g of conditioned weight) litter beds of C. sempervirens–Q. ilex and J. thurifera–Q. faginea were tested. The FMC of samples was fixed at 10–12%, as obtained in the air-conditioned chamber, as previously mentioned. An electric radiator (epiradiator) (power 500 W, heated to 420°C) was used to ignite a standardized pine wood block (2 cm × 2 cm × 1 cm; **Supplementary Figure A3a**) used as point-ignition source to simulate a standard firebrand (Ganteaume et al., 2009) (**Supplementary Figure A3b**).

**(b2)** Linear Fire Propagation tests were performed on litter beds of pure samples of each species and 50% mixtures of C. sempervirens–Q. ilex and J. thurifera–Q. faginea (g/g of conditioned samples). A cotton wick soaked in ethanol was placed at the margin of the litter bed and ignited with a lighter (**Supplementary Figure A3c**).

The following parameters were recorded: litter bed ignition success in an ember (IPe) and a lateral fire-line (IFL) (as percentage of ignition success over 10 attempts), fire propagation capacity with a lateral fire-line (PFL) (percentage of fire propagation success on 10 attempts), and mass consumption after both type of tests (see **Table 2**). In order to assess the ignition and propagation success, each combination of litter bed (pure and mixed) was replicated 10 times. Both types of tests were performed under controlled laboratory conditions (T = 20 ± 2°C, RH = 30 ± 5%).

**(c) Flammability tests and fire resistance of barks**

**FIGURE 2:**
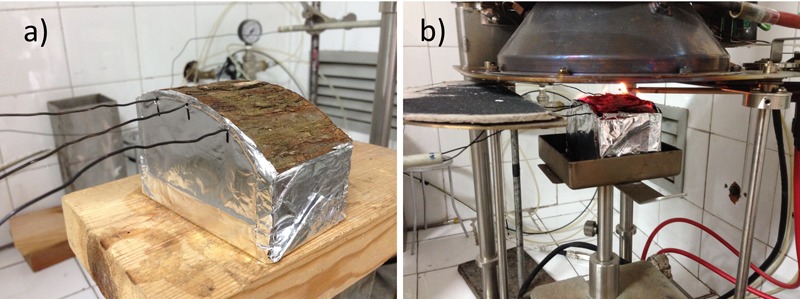
Flammability tests of bark of the four species considered were performed by MLC on 10 cm × 10 cm × 10 cm disk portions **(b)**. Temperature reached at the bark surface and in the cambium were measured with thermocouples **(a)**.

The MLC device, arranged and established as described above, was used according to the methodology proposed by Dehane et al. (2015). A sample of bark and wood (10 cm × 10 cm × 10 cm), with the lateral sides protected by aluminum foil, was inserted in a porous sample holder (**Figure 2**) and placed at a distance of 25 mm from the heater, to simulate an incident heat flux of 25 kW/m^2^ at the top of the sample. Sample ignition was piloted with a sparkle generator. The aluminum foil prevented heat loss through the sides of the sample. Type K thermocouples (diameter 1 mm) were positioned (i) on the surface of the sample (bark surface) (1 thermocouple) and (ii) in the cambium (3 thermocouples homogeneously positioned along the sample) to provide an estimation of the heating rate transmission in the living tissues (**Figure 2**). The temperatures reached during the tests were recorded with a Datataker DT500^®^ data logger. The flammability parameters determined with the MLC are listed in **Table 2**.

The tests were repeated to yield three replicates per species that complied with repeatability criteria (N = 12) according to Madrigal et al. (2009, 2013) and Dehane et al. (2015). Tests were considered complete when the temperature of the cambium reached the temperature measured at the bark surface as it was assumed that the entire bark was burned at this time. According to Dehane et al. (2015), a “fire resistance index” was estimated on the basis of the time to reach 60°C in the inner bark, as an approximation of the vulnerability of living tissues (cambium). The bark moisture content was previously measured on paired samples of each species, as described above (**Table 1**).

### Variables and Statistical Analysis

The flammability variables measured during the tests are shown in **Table 2**, following the classification of flammability components proposed by White and Zipperer (2010). Results concerning live foliage and litter derived from different mixtures and results from bark tests were compared by one-way ANOVA and Tukey HSD test for *post hoc* comparisons, after estimation of the parametric requirements for all variables. When the data did not fulfill the assumptions of parametric tests, logarithmic and angular transformations were carried out. Three replicates per mixture and species/stand complying with the repeatability criteria were included in the ANOVA (*N* = 15 for Jt-Qf; *N* = 15 for Cs-Qi; *N* = 12 for barks).

The correlation between analyzed variables and the proportion of conifers in the sample (percentage of fresh weight of *Cs* and *Jt*) was determined using a non-parametric Spearman test (*p* < 0.05), for a proportion of 100% in unmixed conifer samples and 0% in unmixed *Quercus* (*Qi* and *Qf*) samples (*N* = 15), to detect any relationship between flammability parameters and the sample mixture (live foliage and litter). According to flammability parameters obtained for unmixed samples, the expected values of flammability parameters were calculated for mixed samples, as in van Altena et al. (2012), weighted by the proportion of each species in the mixed sample (25–75%; 50–50%; 75–25%). In addition, a non-parametric Spearman correlation matrix was used to test the relationships between bark flammability parameters and bark thickness (*N* = 12).

The expected values (additive effect) were compared with observed values for each flammability parameter by using a non-parametric Wilcoxon matched pairs test (*p* < 0.05), to detect significant non-additive effects of species in the mixtures. Observed and expected values were plotted in *x*- and *y*- axis to highlight their deviation from linearity.

For litter bed point-ignition tests, a logistic model was fitted including a series of tests with *Pinus pinea* L. needles (100% of ignition success; positive control) to obtain the minimum bulk density required to achieve ignition success in mixed and pure litter beds (*N* = 70).

The devices and types of samples used, the flammability components considered and the variables obtained in the present study are summarized in **Table 2**.

## Results

### Flammability of Pure and Mixed Live Foliage and Litter. Mass Loss Calorimeter Experiments

#### Live Foliage (See **Table 2** for Acronyms)

**FIGURE 3:**
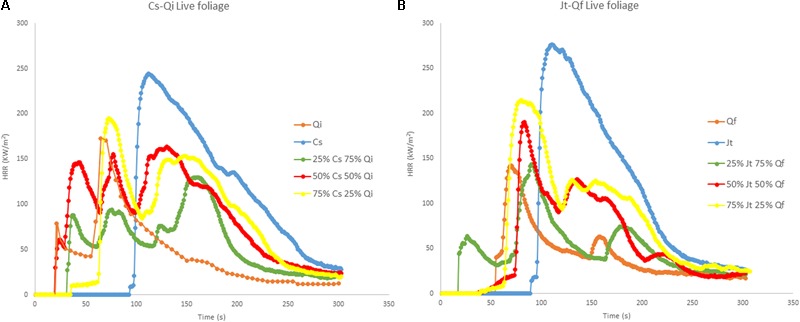
Heat Release Rate (HRR) curves (MLC tests at 50 kW/m^2^) of live foliage: **(A)** pure samples of *C. sempervirens* (*Cs*) and *Q. ilex* (*Qi*), and mixtures of *Cs-Qi*; **(B)** pure samples of *J. thurifera* (*Jt*) and *Q. faginea* (*Qf*) and mixtures of *Jt-Qf*. The tests for the mixtures were carried out for three different proportions of the two mixed species (25/75; 50/50; 75/25).

Flammability tests on live foliage showed that ignitability of *C. sempervirens* (*Cs*) and *J. thurifera* (*Jt*) was lower (higher time to ignition – TTI) than that of *Q. ilex* (*Qi*)and *Q. faginea (Qf)*, respectively (**Figure 3**). Both *Cupressaceae* species (*Cs* and *Jt*) were more combustible (significantly higher peak heat release rate - PHRR) than both *Quercus* species, and of the species considered, *Qf* exhibited the lowest combustibility (lowest PHRR) (**Figure 3**). Interestingly the heat release rate (HRR) curves for the mixtures of both stands were multimodal (multiple ignitions), and the released energy often peaked after the first ignition.

Pure live foliage of *Cs* showed the highest TTI (77.3 s) and the corresponding value was significantly lower in mixed samples with 50% or more *Qi*, while the lowest TTI was recorded in pure live foliage of *Qi* and in the 75% *Qi* mixture (18 and 18.3 s respectively). Similar results were obtained for average effective heat of combustion (AEHC), which was higher in pure *Cs* samples (19.8 MJ/kg) and significantly lower in the 50% mixture with *Qi* (12.5 KJ/kg), and for PHRR, which was the highest in pure *Cs* samples (237.7 kW/m^2^), significantly lower in the 75% *Cs* mixture (186.3 kW/m^2^) and even lower (119.6 kW/m^2^) in the 25% *Cs* mixture. while it was intermediate in pure *Qi*, samples (166.8 kW/m^2^) (**Table 3**). Total heat release (THR) was the highest in *Cs* (24.3 MJ/m^2^) and was significantly lower in the 25% *Cs* mixture (10.9 MJ/m^2^) and in pure *Qi* samples (11 MJ/m^2^). No differences were noted concerning the mass loss rate (MLR) among the different mixtures of the two species, while it was significantly higher in 100% *Qi* samples than in the 100% *Cs* samples (**Table 3**).

**Table 3:** Flammability results of MLC tests on live foliage (*N* = 30).

	*Cupressus sempervirens – Quercus ilex* stand					*Juniperus thurifera – Quercus faginea* stand				
	100*Cs*	100*Qi*	25*Cs*/75*Qi*	50*Cs*/50*Q*i	75*Cs*/25*Qi*	100*Jt*	100*Qf*	25*Jt*/75*Qf*	50*Jt/*50*Qf*	75*Jt*/25*Qf*
TTI	77.3	18.3	18.0	23.0	54. 7	68.3	69.3	51.3	67.3	50.3
*sd*	(23.5)	(2.9)	(10.4)	(12.2)	(18.3)	(21.5)	(10.7)	(64.7)	(4.0)	(8.9)
	a	b	b	b	ab	a	a	a	a	a
PHRR	237.7	166.8	119.6	163.9	186.3	247.6	147.8	142. 0	207.6	217.8
*sd*	(9.8)	(10.3)	(9.5)	12.9	(9.0)	(25.4)	(18.0)	(10.9)	(15.1)	(11.6)
	a	b	c	b	b	a	b	b	a	a
AEHC	19.8	8.2	7.4	12.5	16.2	17.5	7.9	12.4	13.2	14.6
*sd*	(1.9)	(2.0)	(1.9)	(2.2)	(1.1)	(2.8)	(0.7)	(6.0)	(2.2)	(2.4)
	a	cd	d	bc	ab	a	b	ab	ab	ab
THR	24.3	11.0	10.9	19.8	20.5	22.5	7.1	13.2	13.9	19.2
*sd*	(1.4)	(5.0)	(1.8)	(3.0)	(1.5)	(0.7)	(4.9)	(3.1)	(3.6)	(3.9)
	a	b	b	a	a	a	b	ab	ab	a
MLR	0.08	0.12	0.09	0.09	0.07	0.09	0.13	0.07	0.09	0.10
*sd*	(0.01)	(0.02)	(0.00)	(0.00)	(0.01)	(0.01)	(0.04)	(0.02)	(0.02)	(0.01)
	a	b	ab	a	a	a	a	a	a	a
RMF	10.7	7.3	10.0	11.0	10.7	7.3	8.3	10.0	10.0	8.3
*sd*	(2.1)	(0.6)	(1.0)	(1.0)	(1.1)	(1.1)	(1.1)	(2.0)	(1.0)	(1.2)
	ab	a	ab	b	ab	a	a	a	a	a

*Mean values and standard error (sd, in brackets) are shown. Different letters indicate significant differences between live foliage samples in each stand (*C. sempervirens–Q. ilex* and *J. thurifera–Q. faginea*) (Tukey HSD test, *p* < 0.05). The variables are as in **Table 2**. *Cs, Cupressus sempervirens; Qi, Quercus ilex; Jt, Juniperus thurifera; Qf, Quercus faginea;* XX*Cs*/XX*Qi* and XX*Jt*/XX*Qf* are the different proportions (%) of mixture (in fresh weight) for studied species in both stands.*

In the other stand (*Jt–Qf*), there were no differences in TTI, MLR and residual mass fraction (RMF) between pure samples of the two species. THR and AEHC were significantly higher in pure *Jt* (22.5 MJ/m^2^ and 17.5 MJ/kg, respectively) than in pure *Qf* (7.1 MJ/m^2^ and 7.9 MJ/kg, respectively), and the various mixtures of the two species were significantly different from both pure *Jt* and pure *Qf* samples. The PHRR was highest in *Jt* samples and was significantly lower only in the 25% *Jt* mixture (142 kW/m^2^) (**Table 3**).

In summary, PHRR, AEHC, and THR were positively correlated with the percentage of both *Jt* and *Cs* (ρ_s_ = 0.755, 0.895, 0.884 for *Cs*; 0.873, 0.753, 0.851 for *Jt*, respectively) in the mixtures (non-parametric Spearman correlation, *p* < 0.05). The proportion of *Cs* in the mixture with *Qi* (ρ_s_ = 0.765 and 0.541 respectively) was also positively correlated with TTI and RMF, but negatively correlated with MLR (ρ_s_ = -0.851) (**Table 4**).

**Table 4:** Non-parametric Spearman correlation (ρ_s_) between the proportion of the conifer species (*C. sempervirens* and *J. thurifera*) and the flammability variables of the live foliage mixtures (tests at MLC at 50 kW/m^2^, FMC = 78–101%) in both stands (*N* = 15).

Live foliage	FMC	TTI	PHRR	AEHC	THR	MLR	RMF
% *C. sempervirens*	**0.872**	**0.765**	**0.775**	**0.895**	**0.884**	**-0.851**	**0.541**
% *J. thurifera*	0.400	-0.180	**0.873**	**0.753**	**0.851**	-0.120	-0.346
% conifer	**0.556**	**0.365**	**0.814**	**0.828**	**0.861**	**-0.436**	0.149

*The trend observed for the two grouped conifers is also shown (*N* = 30). Significant correlations (*p* < 0.05) are highlighted in bold type.*

**FIGURE 4:**
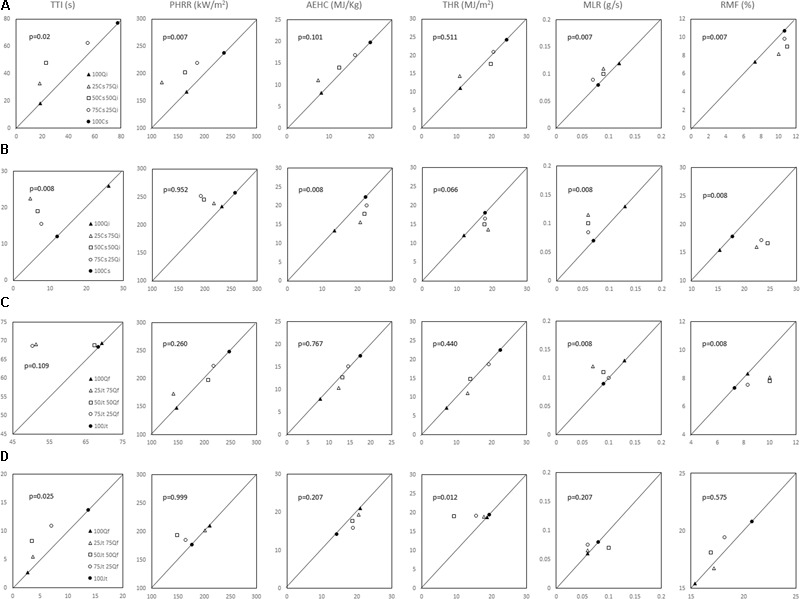
Observed versus expected values for the flammability parameters considered for both live foliage and litter mixtures were evaluated by Wilcoxon’s paired test. *p*-values are reported in each graph **(A)** live foliage of *C. sempervirens–Q. ilex* mixtures; **(B)** litter of *C. sempervirens–Q. ilex* mixtures; **(C)** live foliage of *J. thurifera–Q. faginea* mixtures; **(D)** litter in *J. thurifera–Q. faginea* mixtures. *Cs, Cupressus sempervirens; Qi, Quercus ilex; Jt, Juniperus thurifera; Qf, Quercus faginea*.

Concerning the *Cs–Qi* stand, ignitability (TTI) of live foliage mixtures was significantly higher than expected, whereas combustibility (PHRR) and consumability (MLR and RMF) were significantly lower than expected, exhibiting a non-additive effect of the mixture (Wilcoxon test; *p* ranging from <0.01 to 0.02). In the *Jt–Qf* stand the non-additive effect was only observed in the consumability parameters MLR and RMF which were lower than expected (**Figure 4**). Ignitability and combustibility of *Cs–Qi* mixtures was closer to those of *Q. ilex*, unless its proportion was relatively small, and the transition seemed to be around 25%.

#### Litter

**FIGURE 5:**
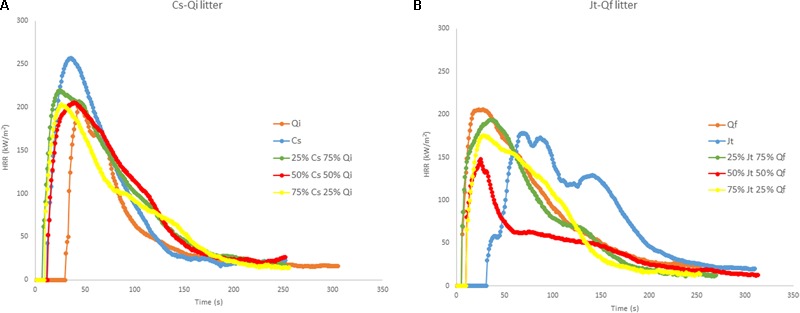
Heat release rate (HRR) curves (MLC tests at 50 kW/m^2^) of reconstructed litter of constant bulk density (20 kg/m^3^): **(A)** pure *C. sempervirens* (*Cs*) and *Q. ilex (Qi)*, and mixtures of *Cs-Qi*; **(B)** pure *J. thurifera (Jt)* and *Q. faginea (Qf)* and mixtures *Jt–Qf*. The tests with mixtures were carried out with three different proportions of the two mixed species (25/75; 50/50; 75/25).

Heat release rate curves for pure and mixed litter samples from the two stands for constant bulk density (20 kg/m^3^) are shown in **Figure 5**. Only slight differences were observed between the HRR curves of pure and mixed litters of *Cs–Qi*: the PHRR was highest in *Cs*, while the TTI was highest in *Qi*. Regarding the *Jt–Qf* stand, the most notable finding was the high TTI in *Jt* (**Figure 5**).

For the *Cs–Qi* stand, the TTI was significantly higher (26.0 s) and the AEHC (13.4 MJ/kg) and THR (12.0 MJ/m^2^) were significantly lower for the *Qi* litter than for pure *Cs* and for all the mixtures with *Cs* litter. PHRR and RMF did not differ significantly between pure and mixed litter samples of *Cs* and *Qi* (**Table 5**). However, for the *Jt–Qf* stand, the TTI (13.7 s) was highest for pure *Jt* litter, and it was significantly lower in the 50% mixture with *Qf* and even lower in pure *Qf* litter (2.7 s). The AEHC was significantly lower in the pure *Jt* litter (14.2 Mj/kg) than in the other types of litter. The PHRR value was highest for pure *Qf* litter and lowest for the 50% mixture of *Jt–Qf*. Neither THR nor MLR differed significantly between any of the pure or mixed litter samples examined. RMF reached the highest value in pure *Jt* samples (20.8%) and reached the lowest value in *Qf* pure samples (15.4%) (**Table 5**).

**Table 5:** Flammability results obtained by MLC tests on litter (*N* = 30).

	*Cupressus sempervirens – Quercus ilex* stand					*Juniperus thurifera – Quercus faginea* stand				
	100*Cs*	100*Qi*	25*Cs*/75*Qi*	50*Cs*/50*Qi*	75*Cs*/25*Qi*	100*Jt*	100*Qf*	25*Jt*/75*Qf*	50*Jt*/50*Qf*	75*Jt*/25*Qf*
TTI	12.0	26.0	4.7	6.7	7.7	13.7	2.7	3.7	3.5	7.0
*sd*	(5.3)	(7.0)	(1.5)	(2.9)	(1.1)	(3.1)	(1.5)	(1.2)	(0.7)	(5.0)
	b	a	b	b	b	a	b	b	b	ab
PHRR	257.7	232.5	217.9	198. 6	192.2	176.6	210.4	201.8	148.6	164.3
*sd*	(21.9)	(41.7)	(18.1)	(19.5)	(9.4)	(3.7)	(14.7)	(21.5)	(1.6)	(12.5)
	a	a	a	a	a	bc	a	ab	c	abc
AEHC	22.3	13.4	20.9	22.0	22.6	14.2	21.0	20.5	18.7	18.9
*sd*	(1.1)	(2.0)	(0.8)	(1.6)	(3.3)	(1.1)	(0.3)	(1.1)	(0.5)	(0.8)
	a	b	a	a	a	b	a	a	a	a
THR	18.0	12.0	18.9	17.9	18.0	19.4	18.7	17.8	19.3	15.6
*sd*	(2.3)	(2.0)	(1.4)	(1.4)	(2.2)	(1.4)	(0.4)	(0.24)	(1.0)	(0.9)
	a	b	a	a	a	a	a	a	a	a
MLR	0.07	0.13	0.06	0.06	0.06	0.08	0.06	0.06	0.10	0.06
*sd*	(0.01)	(0.02)	(0.00)	(0.00)	(0.00)	(0.00)	(0.00)	(0.01)	(0.07)	(0.01)
	a	b	a	a	a	a	a	a	a	a
RMF	17. 8	15.4	22.4	24.5	23.3	20.8	15.4	17.2	16.9	18.2
*sd*	(5.4)	(5.4)	(4.3)	(1.2)	(4.5)	(0.8)	(3.0)	(1.4)	(1.2)	(1.7)
	a	a	a	a	a	a	b	ab	ab	ab

*Mean values and standard error (sd, in brackets) are shown. Different letters indicate significant differences between litter samples in each type of forest (*C. sempervirens–Q. ilex* stand and *J. thurifera–Q. faginea* stand) (Tukey HSD test, *p* < 0.05). Variables are as described in **Table 2**. *Cs, Cupressus sempervirens; Qi, Quercus ilex; Jt, Juniperus thurifera; Qf, Quercus faginea;* XX*Cs*/XX*Qi* and XX*Jt*/XX*Qf* are the different proportion (%) of litter mixtures (fresh weight) of the studied species in both stands.*

The correlation between the proportion of *Cs* in the litter mixtures and flammability parameters was significant only for AEHC (ρ_s_ = 0.644) (**Table 6**). However, in the *Jt–Qf* samples the proportion of *Jt* litter was positively related to TTI, MLR and RMF (ρ_s_ = 0.692, 0.560, and 0.637 respectively), and negatively related to PHRR and AEHC (ρ_s_ = -0.632 vs. -0.690) (**Table 6**).

**Table 6:** Non-parametric Spearman correlation (ρs) between the proportion of conifers (*C. sempervirens* and *J. thurifera*) and the flammability variables of the litter mixtures (tests at MLC at 50 kW/m^2^, FMC = 15.5–30%) in both stands (*N* = 15).

Litter	FMC	TTI	PHRR	AEHC	THR	MLR	RMF
% *C. sempervirens*	-1.000	-0.088	0.033	**0.644**	0.393	-0.273	0.142
% *J. thurifera*	1.000	**0.692**	**-0.632**	**-0.690**	-0.049	**0.560**	**0.637**
% conifer	-0.026	0.332	-0.282	0.072	0.167	0.075	0.308

*The trend observed for the two grouped conifers is also shown (*N* = 30). Significant correlations (*p* < 0.05) are highlighted in bold type.*

The TTI values for litter mixtures from both mixed forests were significantly lower than expected (**Figure 4**). Ignitability of *Jt–Qf* litter mixtures was closer to that of *J. thurifera*, unless its proportion was relatively small, and the transition seemed again to be around 25%. In the *Cs–Qi* stand, AEHC and RMF were higher than expected and closer to those of *C. sempervirens* even when its proportion was as low as 25% (non-additive effect; *p* < 0.01). By contrast, the THR was lower than expected in the *Jt–Qf* mixtures, particularly when the proportion of the two species was 50%. In both stands, the observed PHRR was not significantly different from the expected value.

### Flammability of Litter Beds. Fire Bench Experiments

The slight differences observed between the litter samples in the Mass Loss Calorimeter experiment, considering a constant bulk density, suggest the need to test litter beds by simulating more natural conditions in the fire bench. The results showed a low ignition success of all litter beds with both ignition sources used (central ember or lateral fire-line) and null fire propagation, irrespective of the species and the mixtures (**Table 7**), consequently the mass consumption in each test was negligible (data not shown).

**Table 7:** Ignition and propagation success (%) of reconstructed litter beds of 40 cm × 40 cm (Average bulk density ± standard deviation for each litter bed are shown) in fire bench tests (*N* = 10).

Litter bed	Bulk density (kg/m^3^)	Ignition success with ember (IPe)	Ignition success with fire line (IFL)	Propagation success (PFL)
*J. thurifera*	197 ± 0.5	0	0	0
*Q. faginea*	36 ± 1.1	10	20	0
*Q. ilex*	62 ± 1.8	10	10	0
*C. sempervirens*	190 ± 0.7	0	0	0
*J. thurifera/Q. faginea*	52 ± 2.5	0	20	0
*C. sempervirens/Q. ilex*	98 ± 1.3	0	0	0
*P. pinea*	12 ± 0.3	100	100	100

*Results are compared with those obtained for a litter bed of known low bulk density (*P. pinea* litter: unpublished results) as positive control.*

Pure *Jt* and *Cs* litter and the 50% mixture *Cs–Qi* litter beds did not ignite at all. Nevertheless, the ignition success was 20% for *Jt–Qf* mixed litter beds (50–50%) for the fire-line ignition method, while the ignition success was 10% for pure *Qi* both with ember and fire-line ignition. For pure *Qf*, ignition success was 10 and 20% for respectively the ember and fire-line methods (**Table 7**). The logistic model fitted to predict ignition success with fire-line source and using bulk density as predictor (χ = 40.99, -2LL = 70.94, *p* < 0.01; ROC curve c = 0.94) showed that litter beds with a bulk density higher than 60 kg/m^3^ -due to the presence of *Cupressaceae* species- did not ignite.

### Flammability and Fire Resistance of Tree Barks

**FIGURE 6:**
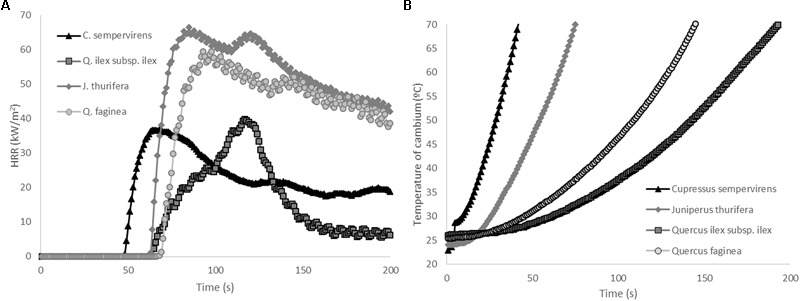
**(A)** Average heat release rate (HRR) curves (kW/m^2^) and **(B)** average temperature increase under the bark (at cambium level) for *C. sempervirens, J. thurifera, Q. ilex*, and *Q. faginea* (MLC tests, radiant heat flux 25 kW/m^2^).

Flammability tests with tree bark samples showed that TTI was highest in *Qi* and lowest in *Cs*, but that the differences were not significant due to the high variability of this trait (**Table 8**). PHRR of bark of *Jt* and *Qf* was significantly higher than that of *Cs* and *Qi*. The flame duration (FD) was lowest in *Qi* samples (90.3 s) and highest in *Qf* samples (1149 s). Both *Cupressaceae* species showed a shorter time of exposure to the heat source was required to reach lethal temperatures at the cambium level (conventionally 60°C) (*Cs* 34 s, *Jt* 57 s) than for the *Quercus* species (125 s for *Qf* and 188 s for *Qi*) (*p* < 0.05, **Figure 6** and **Table 8**), which have a thicker bark (*Cs* = 4 mm, *Jt* = 5.8 mm vs. *Qf* = 9 mm, *Qi* = 16.4 mm) (**Table 1**). Bark thickness was significantly correlated with time to reach lethal temperatures (*ρ* = 0.91, *p* < 0.05). Time to reach lethal temperatures was not significantly correlated with any flammability parameters.

**Table 8:** Flammability parameters and fire resistance of bark, expressed as time to reach 60°C in the cambium (TT_60_) from *C. sempervirens* (*Cs*), *J. thurifera* (*Jt*), *Q. ilex* (*Qi*), and *Q. faginea* (*Qf*). Means and standard deviations (sd, in brackets) are shown.

	*Cs*	*Qi*	*Jt*	*Qf*
TTI (s)	53.8	104.0	55.3	94.5
*sd*	(34.0)	(46.0)	(14.6)	(49.3)
	a	a	a	a
PHRR (kW/m^2^)	34.8	37.2	63.5	58.0
*sd*	(2.6)	(2.4)	(11.4)	(10.5)
	a	a	b	b
FD (s)	521	90.3	551	1149
*sd*	(182)	(24.5)	(164)	(375)
	a	b	a	c
TT_60_ (s)	34	188	57	125
*sd*	(9.7)	(33.6)	(18.0)	(27.9)
	a	c	a	b

*Different letters indicate significant differences in each parameter between species (Tukey HSD test, *p* < 0.05).*

## Discussion

### Flammability of Live Foliage and Litter of Individual Species

In this study, the flammability of the main species in two Mediterranean mixed woodlands was evaluated. The flammability of live foliage and litter samples was first characterized separately for each species, and then for the different mixtures to detect non additive effects (Varner et al., 2015).

Live foliage of *Cs* was less ignitable, and the amount of combusted fuel was lower, but combustion generated more energy for a longer time, as previously reported (Della Rocca et al., 2015). The live foliage of *Jt*, for which no references are available, showed a high combustibility similarly to *Cs*, confirming that this trait appears to be common among *Cupressaceae* (Cornwell et al., 2015).

The flammability of *Cs* and *Qi* was quite different: *Qi* ignited in a shorter time and generated less intense combustion (shorter and lower heat release), but with higher fuel consumption, confirming the typical behavior of species with large leaves (de Magalhães and Schwilk, 2012).

Similar differences were also observed between *Jt* and *Qf*, except for the unexpected low ignitability of the latter species. Though our work was not aimed at evaluating “how live fuels burn,” we can suppose that differences in flammability between species, could depend also on the view factor to radiation source of the respective tested fuels as argued by McAllister et al. (2012) concerning the ignition time.

Smaller differences between the two groups of species were observed in relation to the flammability of litter samples. The litter layer is important for ignition and spread of a surface fire (Ganteaume et al., 2011). The *Qf* litter ignited readily as leaf size and shape (jagged leaf margin) reduced litter packing, thus increasing the oxygen available for combustion (Schwilk and Caprio, 2011). *Qi* litter was surprisingly the less ignitable, possibly due to its thick, tough leaves, which are rich in lignin and take longer to ignite, as reported for *Q. coccifera* (Curt et al., 2011). Moreover, the flattened arrangement of the *Qi* leaves in the sample holder of the MLC device might have altered the transmission of the radiant heat through the sample, increasing the time for ignition. Reduced ignitability of fine forest fuels when submitted to radiant heat flux was experimentally observed by McAllister and Finney (2014). Discrepancy observed at the laboratory scale comparing results from devices using radiant heat (i.e., MLC) to those using convection were partially attributed by these authors to the separation by air space of the typical forest fine fuel and because some particles can block a fraction of radiation.

Usually, litter consisting of larger leaves is more flammable, ignites more quickly and burns faster (giving rise to more heat released and higher flames) than litter comprising fine particles, such as *Cupressaceae* litter (de Magalhães and Schwilk, 2012). The litter of most conifers is considered highly flammable, with the notable exception of the non-*Pinaceae* species, which generally produce dense, non-flammable litter beds (Pinto and Fernandes, 2014; Cornwell et al., 2015). In fact, as already observed in Della Rocca et al. (2015), the ignitability of litter of *Cs* was low.

### Flammability of Live Foliage and Litter Mixtures

Considering the live foliage tests as simulations of crown fire, in the *Cs* and *Qi* mixed samples, we observed that adding *Qi* to the mixture significantly increased ignitability when the amount of *Qi* was between 50 and 75%. The ignitability of mixtures was closer to that of foliage of *Qi* on its own, the more ignitable of the two species, unless its fraction was relatively small, below a threshold of 25%. A similar effect was detected on combustibility, which was closer to the less combustible *Qi* even when its proportion in the mixture was 25%. A clear non-additive effect was indeed evidenced in the live foliage mixture of *Cs–Qi*, as ignitability was higher and combustibility was lower than expected (*p* < 0.02 and 0.007, respectively).

The proportion of *Cs* in the mixtures was positively related to a delay in ignition (higher TTI), intensity of combustion, to sustainability and negatively related to consumability (**Table 6**). Sustainability increased when *Cs* was present in a proportion of 50% or more in the mixture and combustibility increased only when *Cs* was predominant (>75%) due to the strong effect exerted by *Qi*. A non-additive effect was found for consumability which was lower than expected and was closer to that of *Cs*.

The flammability of mixtures is simply predicted by the flammability of the individual species and their proportion in the mixture, as also observed by de Magalhães and Schwilk (2012) for different plant species. The presence of *Qi* had a strong effect, increasing ignitability and reducing the combustibility of the mixtures with *Cs* more than expected; while consumability was lower than expected and was mostly affected by *Cs*. Non-additive effects on crown fuel have not previously been reported, while similar effects were found for litter fuel by van Altena et al. (2012).

As concerning litter mixtures, sustainability was the only parameter which increased significantly with the increase of *Cs* proportion, but no differences were found among the different mixtures of *Cs* and *Qi*. Non-additive effects were recorded for consumability (MLR and RMF), which was lower than expected and for sustainability, which was higher than expected. Both these two parameters were closer to those of *Cs* even when its proportion in the mixture was as low as 25%. Differently to what observed in live foliage, ignitability of pure *Qi* litter samples was lower than both pure *Cs* samples and all the *Cs–Qi* mixtures. The unexpected low ignitability of *Qi* (TTI of 26 s for pure *Qi* decreasing to 4.7 s when *Qi* was 75% in the mixture), probably due to the already mentioned reduced radiant heat flux through the sample in the MLC device, was not found in all the mixtures with *Cs*. The presence of *Cs* fragments intermixed with *Qi* leaves, appeared to favor the transmission of the heat flux through the samples, accelerating the ignition. Differently than live foliage, combustibility of litter mixtures was additively affected.

Regarding the mixed fuels of *Jt* and *Qf*, the combustibility (PHRR) increased as the proportion of Jt increased to 50–75% in the live foliage mixture. A marked reduction in heat released (PHRR) was evident when *Qf* predominated in the mixture (>75%). However, no significant influence in ignitability and combustibility were noted for the different mixtures of the two species. In the *Jt–Qf* live foliage only consumability showed a non-additive effect and was closer to that of *Jt*.

The ignitability of the different mixtures of *Jt-Qf* litters was not significantly different from that of the more ignitable species (*Qf*) and a non-additive effect was recorded (*p* = 0.025). The litter mixtures were less combustible when *Jt* was present in a proportion of at least 50–75%.

If a flammable species is defined as having a very high ignitability and high consumability (van Altena et al., 2012), both *Quercus* species (particularly *Qf*) appeared more flammable than the *Cupressaceae* considered. However, energy release (combustibility) is not necessarily higher in the most flammable species. Indeed, in the present study the highest PHRR and THR were recorded for *Cs* and *Jt*. The total energy released during combustion and the maximum peak may play an important role during fire: high temperatures generated during combustion may speed up the ignition of the surrounding species (van Altena et al., 2012). In this study, we tested mixtures of species characterized as having different levels of flammability: the *Quercus* species exhibiting higher ignitability and the *Cupressaceae*, higher combustibility. The flammability of the litter has been reported to be driven by the most flammable species (de Magalhães and Schwilk, 2012). In both mixed forests under study here, the ignitability and the combustibility of both live foliage and litter generally tended to be driven by oaks, the most flammable component of the mixture, while the consumability and the sustainability tended to be slightly driven by the *Cupressaceae*. A similar effect, described as ‘enhanced dominance,’ has been reported in relation to overall flammability (van Altena et al., 2012).

Our findings suggest that the flammability (particularly ignitability) of a species can influence the temporal dynamics of fire beyond the proportion of its biomass in a mixture. The most ignitable species exerted a strong non-additive effect in the mixture, thus increasing its ignitability. Similarly, the less consumable species reduced the mass loss of the mixtures during combustion. The RMF values of all the mixtures were higher than that of each of the individual species, also exerting a significant non-additive effect. The interaction between species in the mixture may lead to a reduction in the amount of combusted fuel.

### Litter Bed Ignition Tests

The projection of flaming or glowing particles (embers) at a distance from the fire front may generate secondary fires beyond the perimeter of a wildfire. The spot fire ignition of forest beds originates from deposition of smoldering or flaming firebrands on the recipient fuel. This complex process depends on the characteristics of both burning particle (size, state, temperature, energy) and receiving fuel (moisture, temperature, density, porosity) as well as the particle landing traits (full or partial embedding, bouncing, splashing) and the environment (temperature, humidity, wind) (Guijarro et al., 2002; Ganteaume et al., 2009; Urban et al., 2015; Song et al., 2017; Wang et al., 2017).

In our study, ignition of litter beds was investigated by limiting the number of variables and using a flaming wooden cube of constant mass as ignition primer in a laboratory environment, in absence of wind. Conditioned litter fuel samples (10–12% FMC) were also used due to the variability in their moisture content (van Altena et al., 2012; Blauw et al., 2015).

Our results indicated an extremely low ignition success in both the pure and mixed litter beds, mainly due to their high bulk density. Previous studies have demonstrated that litter bulk density is an important factor driving differences in litter flammability (e.g., Ganteaume et al., 2009; de Magalhães and Schwilk, 2012). A dense litter bed can hamper litter fire, even though the material itself is chemically highly flammable (Pinto and Fernandes, 2014; Cornwell et al., 2015; Molina et al., 2017), such as those of *Cupressaceae* species. The results of the fire bench ignition tests corroborate the MLC results, which revealed the lower ignitability of the two *Cupressaceae* species, particularly in *Cs* mixed litters.

The comparison of 100% successful tests with a litter bed with low bulk density (such as *P. pinea* litter) confirmed the relatively low flammability of the litter beds considered under the tested conditions. Fuel density has also been reported to be an important trait explaining the lack of flammability of the litter beds of the *Cupressaceae* by van Altena et al. (2012). Indeed, we also found that ignition success was null either with ember or with fire line beyond a threshold value of 60 Kg/m^3^. A similar transition from flammable to non-flammable litter samples that depended on the packing ratio and density was reported by Cornwell et al. (2015). The results are consistent with those of Ganteaume et al. (2009), who also observed significant negative correlations between point-ignition success and litter bulk density, which was explained by the permeability to the oxygen flow in the litter bed (Varner et al., 2015). The very low ignitability of litter beds we tested may also be affected by the absence of wind during the experiment, which is known to increase the ignition probability, decrease the flaming delay time and increase the smoldering to flaming transition, (Song et al., 2017; Wang et al., 2017).

In the studied mixed forests, the higher proportion of *Cupressaceae* in the litter led to greater interlocking between the fuel elements with small fragments of *Cs* or *Jt* filling the spaces between the larger oak (*Qi* or *Qf*) fuel fragments, thus increasing the resulting density of the mixed litter bed.

### Bark Flammability

Bark is the fundamental protective external part of tree stems, and though there are important differences in thickness and structure between species, the causes of bark diversity are still unclear (Rosell et al., 2014). Studying the effect due to fire to the trunk is useful for predicting the consequences of wildfires on the viability of the trees, as plant tissue mortality due to a fire is largely attributed to the intense heat, which kills the cambium (Bond and van Wilgen, 1996).

The effect of different bark thickness and thermal conductivity due to the rhytidome traits of broadleaf species has been highlighted by Catry et al. (2010) as a factor that may drive sustainable pre-fire and post-fire forest management strategies.

Fire regime and type of fire in particular areas or ecosystems (Johnson and Van Wagner, 1985; Agee, 1994; Sugihara et al., 2006) are essential selective traits for bark thickness of plant species on a global scale (Pausas, 2015).

The species of mixed forests considered in our study belong to different functional groups: two *Quercus* species, *Qi* and *Qf*, with bark of respectively high and medium thickness, and two *Cupressaceae, Cs* and *Jt*, with thin-medium bark and consequently much more sensitive to the high temperatures reached during a wildfire, as the protection afforded to the inner living tissues mainly depends on bark thickness (Frejaville et al., 2013).

Flammable bark should increase fire severity causing necrosis on cambium and the outer sapwood (Dehane et al., 2015). In this study, we observed a slightly negative (non-significant) relationship between bark ignitability and bark thickness (i.e., the greater the thickness, the lower the ignitability), while the combustibility and sustainability appeared independent of this trait. This observation is partly consistent with the findings reported by Frejaville et al. (2013) concerning ignitability; however, these authors observed a positive correlation between bark thickness and the heat released during combustion (combustibility), and Dehane et al. (2015) observed a positive relationship between heat release and lethal temperatures in inner bark in the fire adapted *Q. suber*. The results of the present study suggest that non-fire adapted species (with medium-low bark thickness) provide limited protection to living tissues and lethal temperatures were reached even during the pre-heating phase (*Cupressaceae*) and during the first stages of flaming phase of bark (*Quercus*). The flammability of the bark therefore does not exacerbate/enhance the lethal effect of the heating rate on the cambium. In these non-fire adapted species, the attainment of lethal temperature in the inner living bark is more dependent on the thickness than on the flammability of the bark.

### Adaptation of the Mediterranean Mixed *Cupressaceae*/*Quercus* Stands to Fire

Though the mechanisms whereby Mediterranean oaks are protected against wildfire have been described (relatively thick bark, resprouting ability, etc.) (Catry et al., 2010), little is known about such mechanisms in *Cupressaceae*. Despite frequent exposure to fire, some Mediterranean ecosystems are characterized by thin bark (as the case of *Cupressaceae*) (Pausas, 2015; Rosell, 2016). *Cupressaceae* have been widely distributed throughout the Mediterranean since the Miocene (Mai and Velitzelos, 1997; Kovar-Eder et al., 2006; Sami and Teodoridis, 2013) and have thus probably been frequently exposed to fires in fire prone environments. The survival strategy of the fire sensitive *Cs* and *Jt*, in frequently burned environments, could be similar to that observed by Bowman and Panton (1993) for *Callitris intratropica* in *Eucalyptus* forest in Australia, in which fire spread is prevented by densely packed litter. This is consistent with the high bulk density and consequent low ignitability observed for the litter of the two *Cupressaceae* examined in the present study. Moreover, litter of cypress species does not allow development of a dense and continuous understory due to an assumed allelopathic effect and its slow decomposition rate, resulting in the accumulation of thick layers of plant debris, in addition to the severe shading of the ground exerted by the dense crowns (Della Rocca et al., 2014). Hence, in *Cupressaceae* stands the fuel available for surface fires is reduced.

Furthermore, the crowns of both *J. thurifera* and *C. sempervirens* subsp. *horizontalis*, are characterized by live branches (from the bottom) with green leaves only in the distal portion of the shoots, without much dead foliage remaining on the branches establishing a fuel discontinuity between the crown and trunk. Therefore, in the case of moderate fires, the presence of live fuel at a distance from the trunk may contribute to the survival of the tree cambium. However more *in situ* research is needed to validate these assumptions.

## Conclusion

In contrast to other types of disturbance in which the intensity and frequency are independent of plant and ecosystems properties, in the case of fire, the disturbance itself is shaped by ecosystem and forest species features regarded as fuels, and the resistance of forest communities is related to the flammability of the vegetation and fuels (Bauhus et al., 2017). The flammability of species of two Mediterranean mixed-stands was determined in the present study. Non-additive effects were observed in some flammability components of live foliage and litter, especially in the mixtures of *C. sempervirens* and *Q. ilex*.

The studied species exerted contrasting effects on the flammability of mixtures, increasing ignitability and decreasing combustibility and consumability more than expected based on their proportion in the mixture. This may influence fire dynamics in the mixed forests. Though it’s difficult to predict the landscape-level fire behavior based on the results of small scale species-level experiments, the low ignitability observed for the mixed litter beds (due to the high bulk density of the *Cupressaceae* litter), associated to the limited development of understory in *Cupressaceae* stands, may potentially reduce the occurrence of surface fires in the studied mixed forests. While the presence of *Quercus* could reduce the intensity of fire, mitigating the high combustibility of *Cupressaceae*. Our study is the first to show non-additive effects in the flammability of live foliage mixtures.

## Author Contributions

GDR and JM conceived the idea. GDR, CH, MG, and JM designed the methodology. GDR, JM, and RD collected and analyzed the data and wrote the manuscript. All authors contributed critically to the drafts and gave final approval for publication.

## Conflict of Interest Statement

The authors declare that the research was conducted in the absence of any commercial or financial relationships that could be construed as a potential conflict of interest.

## Supplementary Material

FIGURE A1 Stand structure in the *C. sempervirens-Q. ilex* mixed forest (Florence, Italy) **(A)**, and in the *J. thurifera-Q. faginea* mixed forest (Guadalajara, Spain) **(B**).

FIGURE A2 Shape of twigs with foliage in *Q. ilex* **(C)** and *C. sempervirens* **(B)** and *Q. faginea* **(A)** and *J. thurifera* **(D)**.

FIGURE A3 Litter bed ignition and fire propagation tests were performed on a fire bench using a flaming cube of wood as an ember **(a,b)** and a cotton wick to simulate the advancing surface fire front **(c)**, respectively.

TABLE A1 Geographical and climate characteristics of the sampling sites. Characteristics of the protection mixed forests of *Cupressus sempervirens* and *Quercus ilex* in Monte Morello area, Florence (Italy) and of *Juniperus thurifera* and *Quercus faginea* in Sela, Guadalajara (Spain).
