# Supplementary figures and images for: Flammability of Two Mediterranean Mixed Forests: Study of the Non-additive Effect of Fuel Mixtures in Laboratory
Source: Front Plant Sci. 2018 Jun 25;9:825. doi: 10.3389/fpls.2018.00825 (PMC6036284; doi:10.3389/fpls.2018.00825)

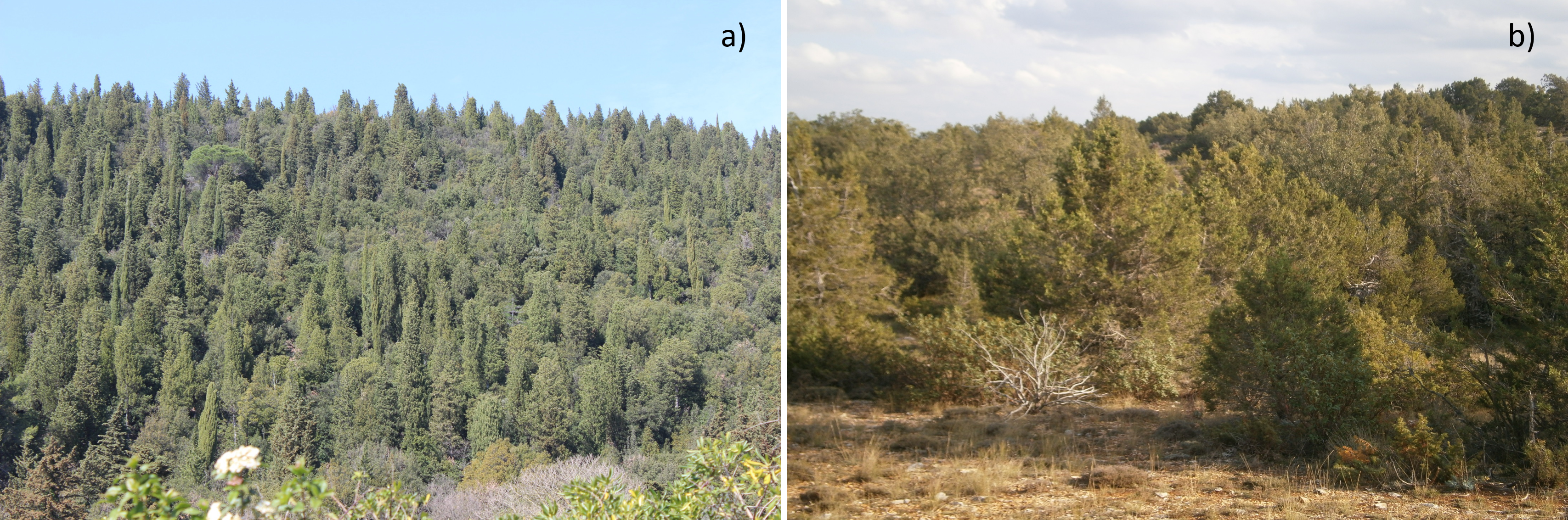

Supplement: FIGURE A1 — Stand structure in the C. sempervirens-Q. ilex mixed forest (Florence, Italy) (A), and in the J. thurifera-Q. faginea mixed forest (Guadalajara, Spain) (B). [file Image_1.TIF]

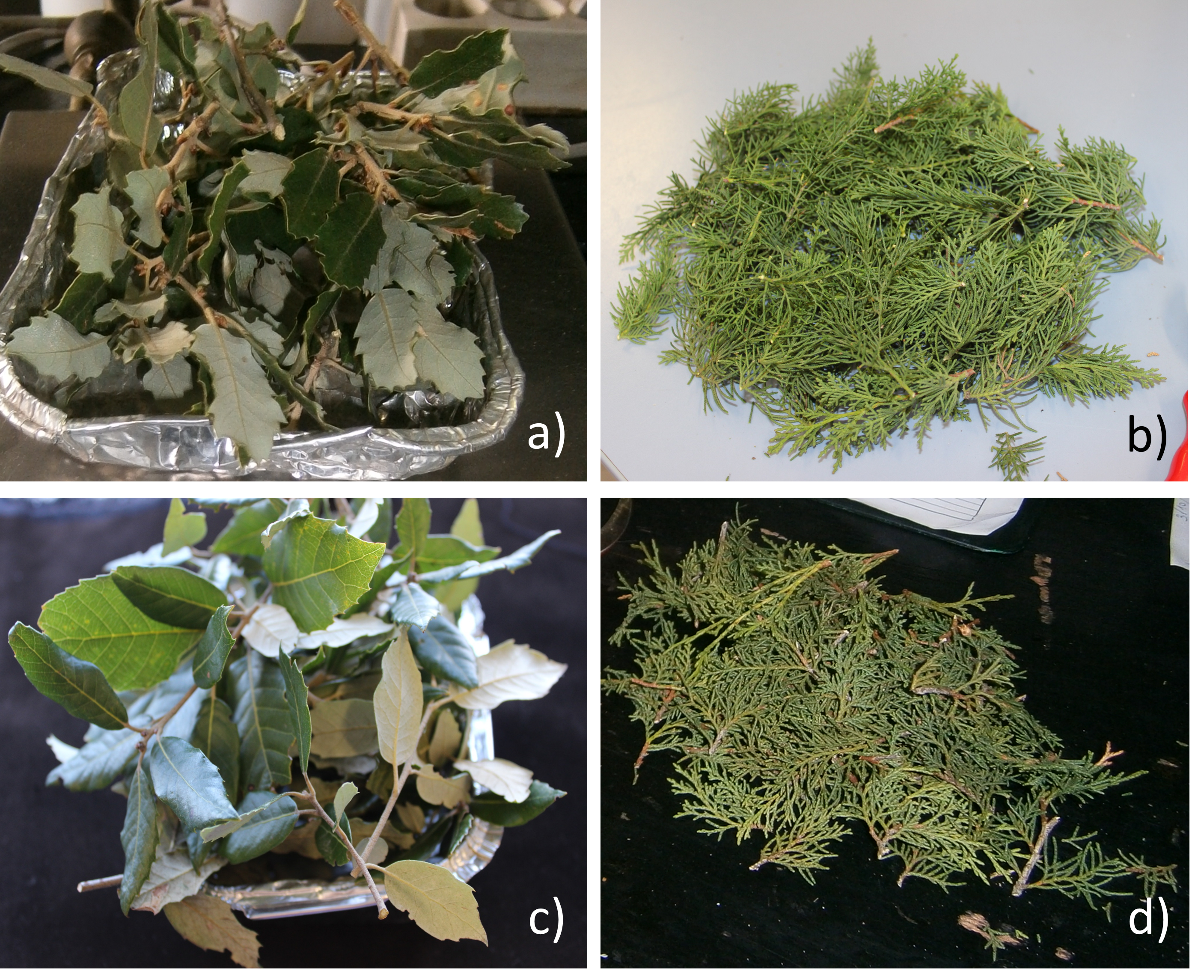

Supplement: FIGURE A2 — Shape of twigs with foliage in Q. ilex (C) and C. sempervirens (B) and Q. faginea (A) and J. thurifera (D). [file Image_2.TIF]

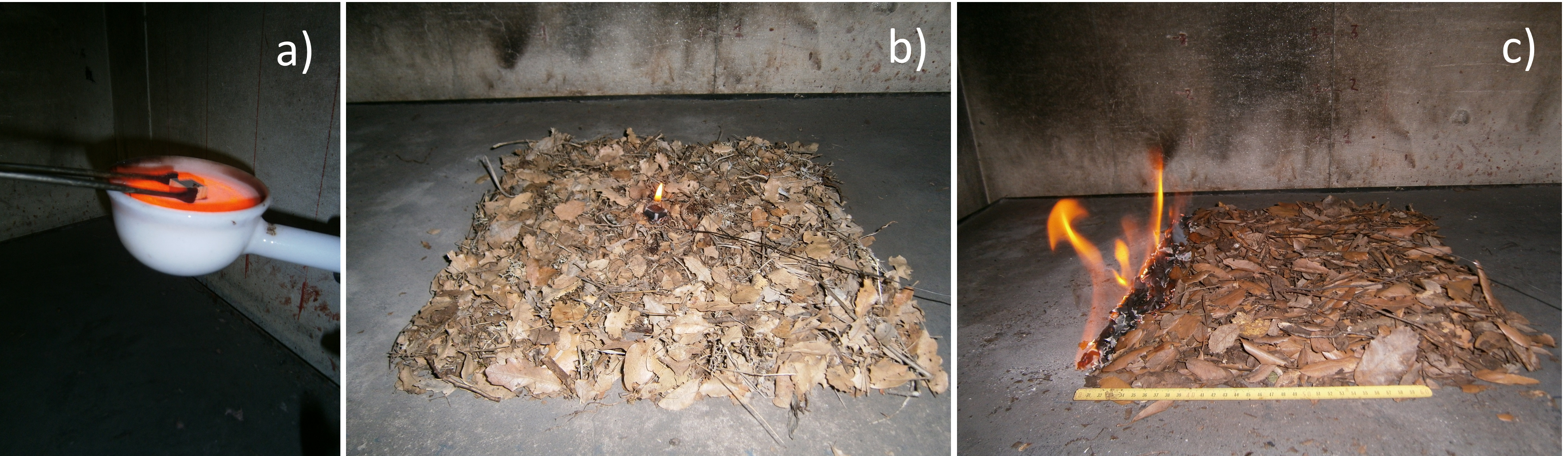

Supplement: FIGURE A3 — Litter bed ignition and fire propagation tests were performed on a fire bench using a flaming cube of wood as an ember (a,b) and a cotton wick to simulate the advancing surface fire front (c), respectively. [file Image_3.TIF]
