# Supplementary material for: Flammability of Two Mediterranean Mixed Forests: Study of the Non-additive Effect of Fuel Mixtures in Laboratory
Source: Front Plant Sci. 2018 Jun 25;9:825. doi: 10.3389/fpls.2018.00825 (PMC6036284; doi:10.3389/fpls.2018.00825)
Supplement: TABLE A1 — Geographical and climate characteristics of the sampling sites. Characteristics of the protection mixed forests of Cupressus sempervirens and Quercus ilex in Monte Morello area, Florence (Italy) and of Juniperus thurifera and Quercus faginea in Sela, Guadalajara (Spain). [file Table_1.DOCX]

**TABLE A1** Geographical and climate characteristics of the sampling sites Characteristics of the protection mixed forests of *Cupressus semprevirens* and *Quercus ilex* in Monte Morello area, Florence (Italy) and of *Juniperus thurifera* and *Quercus faginea* in Sela, Guadalajara (Spain).

| Forest | Location | Coordinates | Mean annual temperature / Annual precipitation | Soil type / vegetation type |
| --- | --- | --- | --- | --- |
| *Cupressus sempervirens - Quercus ilex* | Serpiolle, Florence, Italy | 43°50.1 N  11°14.7 E  Elevation = 302 m.a.s.l. | 14.1 °C  861 mm | Humic Eutrudepts, clayed-skeletal, mixed, mesic / Cypress woodlands with broadleaves |
|  |  |  |  |  |
| *Juniperus thurifera - Quercus faginea* | Selas,  Guadalajara, Spain | 40° 56.1 N  2° 07.3 W  Elevation=1280 m.a.s.l. | 9.7 °C  533 mm | Limestones over dolomites and marls / Open *Juniperus* woodlands with broadleaves |

Dendrometric traits

N. of trees/ha Basal Area/ha Mean Diameter Mean Height

*C. sempervirens* 1447.5 25.6 m^2^ 15.1 cm 10.7 m

*Q. ilex* 499.7 6.7 m^2^ 13.1 cm 6.5 m

*J. thurifera* 234.12 9.01 m^2^ 22.04 cm 5.7 m

*Q. faginea* 937.25 16.61 m^2^ 15.77 cm 6.1 m

Tree social position

Dominant/mature Sub-dominant Dominated or young

*C. sempervirens* 30.7% 26.7% 34%

*Q. ilex* 28% 14.1% 43.5%

*J. thurifera* 14% 52% 34%

*Q. faginea* 42% 37% 21%

Crown length (% of trees showing a crown length expressed as % of total tree height)

Between 60-80% H Between 20-40% H <20% H

*C. sempervirens* 6.7% 45.7% 28.8%

*Q. ilex* 31.8% 30.8% 20.5%

*J. thurifera* 93% 7% 0%

*Q. faginea* 78% 22% 0%

References:

Boschi e macchie di Toscana - L’inventario Forestale (1998). Hofmann et al. (eds.). Edizioni Regione Toscana, centro stampa giunta regionale, Firenze, Italy. Pp. 219.

National Forest Inventory (Ministry of Agricultural, Fishering and Environment of Spain).

<https://it.climate-data.org/location/731898/>;

<http://sit.lamma.rete.toscana.it/websuoli/>;

<http://www502.regione.toscana.it/geoscopio/usocoperturasuolo.html>;

<http://info.igme.es/cartografia/>
